# Comparison of ultrasonography with Doppler and MRI for assessment of disease activity in juvenile idiopathic arthritis: a pilot study

**DOI:** 10.1186/1546-0096-10-23

**Published:** 2012-08-16

**Authors:** Louise Laurell, Michel Court-Payen, Susan Nielsen, Marek Zak, Mikael Boesen, Anders Fasth

**Affiliations:** 1 Department of Pediatrics, Skåne University Hospital, Lund University, Lund, Sweden; 2 Department of Diagnostic Imaging, Gildhøj Private Hospital, University of Copenhagen, Copenhagen, Denmark; 3 Department of Pediatrics, Rigshospital, University of Copenhagen, Copenhagen, Denmark; 4 Department of Radiology, Frederiksberg Hospital, and Parker Institute, University of Copenhagen, Copenhagen, Denmark; 5 Department of Pediatrics, University of Gothenburg, Gothenburg, Sweden

**Keywords:** Ultrasonography, Color Doppler, MRI, Juvenile idiopathic arthritis

## Abstract

**Background:**

In juvenile idiopathic arthritis (JIA), the trend towards early therapeutic intervention and the development of new highly effective treatments have increased the need for sensitive and specific imaging. Numerous studies have demonstrated the important role of MRI and US in adult rheumatology. However, investigations of imaging in JIA are rare, and no previous study has been comparing MRI with Doppler ultrasonography (US) for assessment of arthritis. The aim of the present study was to compare the two imaging methods regarding their usefulness for evaluating disease activity in JIA, and to compare the results with those obtained in healthy controls.

**Methods:**

In 10 JIA patients (median age 14 years, range 11–18), 11 joints (six wrists, three knees, two ankles) with arthritis were assessed by color Doppler US and MRI. The same imaging modalities were used to evaluate eight joints (three wrists, three knees, two ankles) in six healthy age- and sex-matched controls. The US examinations of both the patients and controls were compared with the MRI findings.

**Results:**

In 10 JIA patients, US detected synovial hypertrophy in 22 areas of 11 joints, 86% of which had synovial hyperemia, and MRI revealed synovitis in 36 areas of the same 11 joints. Erosions were identified by US in two areas of two joints and by MRI in six areas of four joints. Effusion was shown by US in nine areas of six joints and by MRI in 17 areas of five joints. MRI detected juxta-articular bone marrow edema in 16 areas of eight joints.

**Conclusions:**

The results of this pilot study indicate that both MRI and US provide valuable imaging information on disease activity in JIA. Importantly, the two techniques seem to complement each other and give partly different information. Although MRI is considered to be the reference standard for advanced imaging in adult rheumatology, US seems to provide useful imaging information that could make it an option in daily clinical practice, in JIA as well as in adult rheumatology. However, the current work represents a pilot study, and thus our results need to be confirmed in a larger prospective clinical investigation.

## Background

Considering juvenile idiopathic arthritis (JIA), the trend towards early therapeutic intervention and the development of new highly effective treatments have probably improved the outcome in many cases, but they have also increased the need for sensitive and specific methods to evaluate disease activity. According to recent investigations, clinical examination alone is inadequate to identify structures involved in JIA, and US often detects subclinical synovitis [1,2], especially in the hands and feet [3,4]. Clinical assessment of disease activity in the small joints of the hand is particularly prone to disagreement among clinicians [5] and the foot and knee are the most frequently involved joints in JIA.

In adult rheumatology, numerous studies have established the important role of MRI [6] and US [7] in assessment of disease activity, and MRI is considered the reference standard for advanced imaging. Nevertheless, due to differences in disease characteristics and the unique features of the growing skeleton, the findings of studies in adults are not directly applicable to children and adolescents [8] and thus far only a few MRI studies of JIA have been conducted, all of which have used different methodologies [9-12]. Modern imaging techniques have not been used to their full potential in pediatric rheumatology [10,13], and imaging studies in this context are still rare. Nonetheless, four recent investigations of JIA have shown that US and Doppler US are more sensitive in the detection of disease activity than clinical examination alone [1-4].

US offers specific advantages over MRI in that it is non-invasive, does not require sedation or general anesthesia, is quickly accessible bedside, and is easy to combine with clinical assessment. Agitation of the patient is rarely a problem, which means that young children can be seated on a parent’s lap or play while being examined, and assessment of multiple locations is possible during a single session. Furthermore, modern high-frequency US transducers, in the hands of an experienced US examiner, provide unsurpassed resolution of the superficial musculoskeletal structures in children.

The aim of the present pilot study was to compare MRI and US with Doppler regarding their value in assessing the different aspects of disease activity in JIA, and to compare the results with images obtained in healthy controls.

## Methods

A descriptive study with controls was conducted from 2007 to 2011 at the Department of Pediatrics, University of Copenhagen, Denmark. Consecutive patients, under the age of 18 years with clinically active arthritis (i.e., swelling or a limited range of joint movement with joint pain or tenderness) in the wrist region, knee, or ankle region, seen at the Pediatric Rheumatology Outpatient Clinic, Rigshospitalet, Copenhagen, Denmark, were invited to participate.

Eleven JIA patients with 12 clinically active joints consented to take part and were assessed by US and MRI. After the patient cohort had been established, age- and sex-matched controls were recruited among young healthy volunteers who had no history of arthritis or chronic pain; these individuals belonged to families of hospital staff members. The controls were assessed at the Department of Radiology, Skåne University Hospital, Malmö, Sweden, using the same US equipment, the same type of MRI equipment and the same protocols as for the patients in Denmark. One healthy 6-year-old control was excluded because she could not remain still during the MRI examination, and hence the matched patient was also excluded. Thus ten patients (median age 14 years, range 11–18 years) with 11 affected joints (six wrists, three knees, two ankles) and six healthy controls with eight joints (three wrists, three knees, two ankles) were left for evaluation. Patient characteristics and pharmacological treatments are summarized in Table 1. Seven of the ten patients (70%) were female. Five had polyarticular (poly) JIA, two oligoarticular (oligo) JIA, one enthesitis-related arthritis, one psoriasis arthritis, and one systemic onset type JIA with polyarticular course.

**Table 1:** Patient characteristics and pharmacological treatment of 10 JIA patients with 11 symptomatic joints

**Characteristics**		**Number (%)**	**Median**	**Range**
Sex				
	Male	3 (30%)		
	Female	7 (70%)		
Joint				
	Wrist	6 (55%)		
	Knee	3 (27%)		
	Ankle	2 (18%)		
Subgroup				
RF-negative polyarthritis		5 (50%)		
Oligoarthritis		2 (20%)		
Enthesitis related arthritis		1 (10%)		
Psoriasis arthritis		1 (10%)		
Systemic		1 (10%)		
Age, years			14	11–18
Disease duration, years			4.3	0.2–10.2
Drug therapies				
	Local steroids	1 (10%)		
	Sulfasalazine	1 (10%)		
	MTX	3 (30%)		
	Etanercept	1 (10%)		
	Golimumab	1 (10%)		
	MTX + etanercept	2 (20%)		
	MTX + golimumab	1 (10%)		

The local research ethics committees approved the study. All parents gave informed consent for their children to participate, and oral assent was obtained from the children themselves.

### Clinical assessment

Patients who had previously been diagnosed with JIA based on the revised criteria of the International League of Associations for Rheumatology (ILAR, 2004) [14], were examined by either of two experienced pediatric rheumatologists for clinical signs of joint involvement.

### US assessment of patients and healthy controls

Patients were assessed clinically and by US on a single day. All the US examinations of patients and controls were conducted by the same experienced radiologist (MCP) specialized in musculoskeletal US. B-mode US was performed to detect structural abnormalities (i.e., synovial hypertrophy or effusion in the joint recesses or bone erosions), and color Doppler was used to identify synovial hyperemia. The Outcome Measures in Rheumatology Clinical Trials (OMERACT) definitions for US pathology (joint effusion, synovial hypertrophy, bone erosions and tenosynovitis) in RA [15] were used. US and color Doppler examinations were performed with a Logiq 9 US-scanner (GE Healthcare, Chalfont St. Gilles, UK) equipped with a 16-4 MHz linear transducer. Color Doppler settings were standardized as follows: the pulse repetition frequency (PRF) was 0.6 KHz, the color Doppler gain was set just below the level at which noise appeared, and the wall filter was very low (65 Hz). The findings of the color Doppler examination were assessed as presence or absence of hyperemia.

Four different anatomical locations (areas) were examined in the knee and ankle and three different areas in the wrist (Tables 2 and 3). US examinations of the knee were carried out with the subject in supine position and the knee in slight flexion and resting on a small pad. Anteriorly, the supra- and parapatellar recesses were scanned in sagittal and axial planes. The medial and lateral femoro-tibial joint recesses were scanned in coronal planes. Finally, a dorsal US examination in axial and sagittal planes was carried out with the individual in prone position and the knee in neutral position; this was done to detect a possible Baker’s cyst.

**Table 2:** Pathological findings detected by MRI and US in 11 clinically affected joints

** Joint**	**Synovial hypertrophy**		**Synovial contrast enhancement/hyperemia**		**Effusion**		**Erosion**	
	**MRI**	**US**	**MRI**	**US**	**MRI**	**US**	**MRI**	**US**
Ankles (n = 2)	2	2	2	2	1	2	0	1
Knees (n = 3)	3	3	3	2	3	3	0	0
Wrists (n = 6)	6	6	6	6	1	1	3	1
Total (n = 11)	11	11	11	10	5	6	3	2

**Table 3:** **Pathological findings detected by MRI and US in areas examined**^**1**^**in 11 clinically affected joints**

			**Pathological findings**							
**Joint**	**Areas examined**		**Synovial hypertrophy**		**Synovial contrast enhancement/hyperemia**		**Effusion**		**Erosion**	
		**n**	**MRI**	**US**	**MRI**	**US**	**MRI**	**US**	**MRI**	**US**
Ankle	talo-crural	2	2	2	2	2	2	1	0	1
n = 2	talo-navicular	2	1	1	1	1	1	0	0	0
	post. subtalar	2	2	1	2	1	1	1	0	0
	tendon sheaths	2	1	0	1	0	0	0	n/a	n/a
Knee	anterior	3	3	2	2	2	3	2	0	0
n = 3	lateral	3	3	2	3	2	2	1	0	0
	medial	3	3	3	3	2	2	3	0	0
	Baker’s cyst	3	3	1	3	0	1	1	n/a	n/a
Wrist	radio-carpal	6	6	6	6	6	1	0	3	1
n = 6	midcarpal	6	6	3	6	3	0	0	2	0
	dist. radio-ulnar^2^	6	5	-	5	-	2	-	0	-
	tendon sheaths	6	1	1	1	0	0	1	n/a	n/a
Number of pathological findings		Tot. 44^1^	36	22	35	19	15	10	5	2

^1^44 areas examined by MRI, 38 areas examined by US.

^2^Distal radio-ulnar joint examined by MRI only, not by US.

n/a = non applicable.

US examination of the ankle was performed with the subject in supine position and the ankle in slight plantar flexion. The following synovial recesses were examined: talo-crural joint (anterior recess, sagittal planes), posterior subtalar joint (lateral recess, coronal planes), talo-navicular or anterior subtalar joint (dorsal and medial recesses, longitudinal planes), and medial, lateral, and anterior tendon sheaths (axial planes).

The US examination of the wrist region was done with the subject seated and the hand resting on a small pad. The dorsal radio-carpal and midcarpal recesses were scanned in sagittal planes, and the extensor tendon sheaths in axial planes with the hand in pronation and minimal palmar flexion. The palmar recesses of the radio-carpal and midcarpal joints were displayed in sagittal planes and the flexor tendon sheaths in axial planes, with the hand in a supine position and minimal dorsal flexion.

In all examinations, the total imaging time per joint was approximately 10 to 15 min.

### MRI assessment of patients and healthy controls

Inasmuch as healthy controls were to be included in the study, it was considered unethical to use general anesthesia during MRI for either patients or controls, and therefore only school-age children were eligible for participation. The OMERACT Rheumatoid Arthritis MRI Score (RAMRIS) definitions for MRI pathology [16] were used to assess the wrist regions, and the same definitions for joint pathologies were used for knees and ankles, and adjusted to apply to each joint. The same MRI protocols were used in patients and healthy controls, except that no Gadolinium contrast (Magnevist, Bayer, Germany) was administered to the controls. Detailed descriptions of MRI protocols are included in ‘ Additional file 1’. MRI examinations of patients were done using either a 3.0 Tesla Trio MRI scanner (Siemens, Erlangen, Germany; five joints in four patients) or a 3.0 Tesla Verio MRI scanner (Siemens, Erlangen, Germany; six joints in six patients and all eight joints in the healthy controls).

Knees were examined in the dedicated send-receive 16-channel knee coil (both 3T Trio and Verio). Ankles were examined in a dedicated send-receive ankle coil with a slight plantar flexion. Wrists were examined either using the semiflex four-channel coil with the patient supine and the hand along the side of the body (3T Verio), or with the patient prone in the ‘superman’ position (3T Trio). For each joint the following MRI sequences were performed. Examinations of the knee: a gradient echo scout, coronal and sagittal STIR, sagittal 3D proton density weighted (PDw) FS TSE SPACE, and sagittal gradient echo 3D T1w VIBE. Total imaging time varied between 30 and 40 min. Examination of the ankle: a gradient echo scout, axial and sagittal T1w TSE, coronal and sagittal STIR and sagittal gradient echo 3D T1w VIBE. Total imaging time varied between 30 and 40 min. The examination of the wrist: a gradient echo scout, coronal T1 weighted (T1w) turbo spin echo (TSE), coronal STIR or fat saturated (FS) T2w (3T Trio), axial STIR or FS T2w TSE (3T Trio) covering wrist and MCP joints, and gradient echo 3D T1w VIBE. Total imaging time varied between 25 and 35 min.

In patients, the T1-weighted sequences for all joints were repeated 5 minutes after intravenous injection of 0.1 ml/kg body weight Gadolinium contrast.

### Evaluation of US images

Doppler US images were analyzed on the screen in real time and images were saved as jpg files. Information on the results was saved in an in-house database for later comparison with the MRI findings. All US examinations were scored according to the presence or absence of US pathology, using the OMERACT definitions [15], and hyperemia was defined as any presence of synovial vascularization as revealed by color Doppler examination [17].

### Evaluation of MRI images

Another experienced musculoskeletal radiologist (MB) analyzed all MRI images blinded to the results of the US examinations. Four different anatomical areas were assessed in the knee region, ankle region, and wrist, respectively (Table 3).

All wrist MRI examinations were scored according to the presence or absence of MRI pathology, using the RAMRIS definitions of pathology [16]: synovitis was regarded as above normal post-gadolinium enhancement (signal intensity increase) of a thickness greater than the width of the normal synovium (compared with T1-weighted images) in scans obtained before and after intravenous Gadolinium contrast; bone marrow edema was described as a lesion located within the trabecular bone and displaying ill-defined margins and signal characteristics consistent with increased water content on the STIR or FS T2w images; bone erosion was defined as a sharply marginated bone lesion that showed correct juxta-articular localization and typical signal characteristics (i.e., loss of normal low signal intensity of cortical bone and loss of normal high signal intensity of trabecular bone on T1-weighted images) and was visible in two planes with a cortical break in at least one plane. The same RAMRIS definitions of pathology (synovitis, bone marrow edema, and erosion) were also used for knees and ankles, and adjusted to apply to each joint. In addition, we registered the presence or absence of tenosynovitis, joint effusion, and Baker’s cyst (knees only). The time needed to score each joint varied between 10 and 20 min depending of the amount of pathology, and the fastest scoring was achieved for the healthy controls showing no abnormalities. All information was saved in our in-house database for later comparison with the US findings.

### Comparison of imaging modalities

Forty-four areas were examined by MRI, 38 areas by US (Table 3). We compared the US and MRI results obtained in patients and healthy controls. This was done by evaluating the presence or absence of the following: (1) synovial hypertrophy and/or synovial perfusion (hyperemia on Doppler US and contrast enhancement on MRI in joint recesses and tendon sheaths in patients, and abnormal signal intensity in the STIR or FS T2w MRI images of healthy controls); (2) joint effusion; (3) bone erosions; (4) bone edema in STIR or FS T2w MRI images. We considered conformity to occur (Tables 4 and 5) when the US and MRI results were in accordance with each other. The results in this study are presented as absolute qualitative values without any statistical calculations due to the small size of the sample.

**Table 4:** **Conformity of MRI and US findings in areas examined**^**1**^**in 11 clinically affected joints**

				**Number of areas with conformity**			
	**Joint**	**Area**		**Synovial hypertrophy**	**Synovial contrast enhancement or hyperemia**	**Effusion**	**Erosion**
			**n**				
	Ankle	talo-crural	2	2	2	1	1
	n = 2	talo-navicular	2	0	0	1	2
		post. subtalar	2	1	1	2	2
		tendon sheaths	2	1	1	2	n/a
	Knee	anterior	3	2	1	2	3
	n = 3	lateral	3	2	2	2	3
		medial	3	3	2	2	3
		Baker’s cyst	3	1	0	1	n/a
	Wrist	radio-carpal	6	6	6	5	4
	n = 6	midcarpal	6	3	3	6	4
		dist. radio-ulnar^2^	-	-	-	-	-
		tendon sheaths	6	6	5	5	n/a
Total	11		38^1^	27	23	29	22

^1^44 areas examined by MRI, 38 areas examined by US.

^2^Distal radio-ulnar joint examined by MRI only, not by US.

n/a = non applicable.

**Table 5:** **Conformity of MRI and US findings in areas examined**^**1**^**in 8 joints of healthy controls**

				**Number of areas with conformity**			
	**Joint**	**Area**		**Synovial hypertrophy**	**Synovial contrast enhancement or hyperemia**	**Effusion**	**Erosion**
			**n**				
	Ankle	talo-crural	2	2	2	2	2
	n = 2	talo-navicular	2	2	2	2	2
		post. subtalar	2	2	2	2	2
		tendon sheaths	2	2	2	2	n/a
	Knee	anterior	3	3	3	2	3
	n = 3	lateral	3	3	3	2	3
		medial	3	3	3	2	3
		Baker’s cyst	3	3	3	3	n/a
	Wrist	radio-carpal	3	3	3	2	3
	n = 3	midcarpal	3	3	3	3	3
		dist. radio-ulnar^2^	-	-	-	-	-
		tendon sheaths	3	3	3	2	n/a
Total	8		29^1^	29	29	24	21

^1^32 areas examined by MRI, 29 areas examined by US.

^2^Distal radio-ulnar joint examined by MRI only, not by US.

n/a = non applicable.

## Results

### Patients

**Figure 1:**
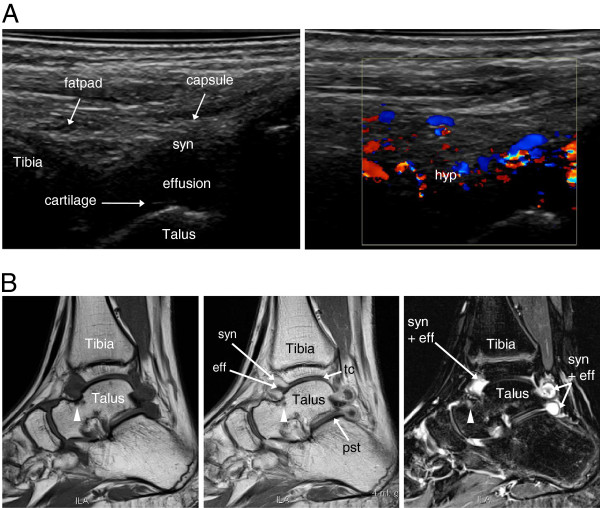
**Imaging of the symptomatic ankle region of an 11-year-old girl with JIA.** (**A**) Sagittal US scanning of the anterior talo-crural joint showing synovial hypertrophy (syn) to the left and hyperemia (hyp) on color Doppler to the right. (**B**) T1-weighted sagittal MRI images before (left) and after (middle) contrast injection. The left image reveals a small bony erosion at the neck of the talus (arrowhead). The erosion is surrounded by bone marrow edema (arrowhead, middle image). In the same image synovial hypertrophy with contrast enhancement (syn) and effusion (eff) are visualized in the anterior and posterior recesses of the talo-crural (tc) and posterior subtalar (pst) joints. On the STIR image to the right, high signal intensity depicts the location of synovitis (syn + eff) and the bone marrow edema surrounding the erosion (arrowhead, right image).

**Figure 2:**
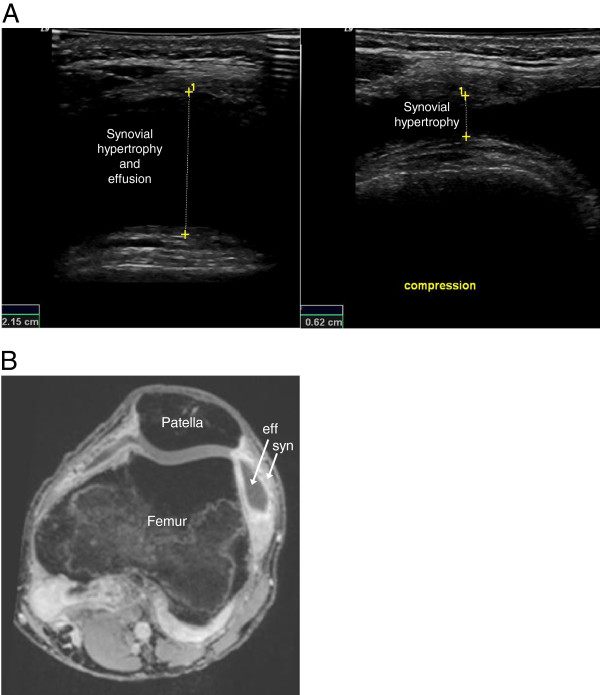
**Imaging of the symptomatic and swollen knee of a 17-year-old boy with JIA.** (**A**) Axial US scanning lateral to the patella. The left image shows an anechoic recess composed of synovial thickening and effusion, measured (1) before compression. In the right image, compression of the recess has transposed the effusion and enabled measurement (1) of the hypertrophic synovium. (**B**) A 3D T1 gradient echo VIBE MRI image of the same joint showing the enhanced hypertrophic synovial tissue (syn), and the effusion (eff), following intravenous injection of Gadolinium contrast.

**Figure 3:**
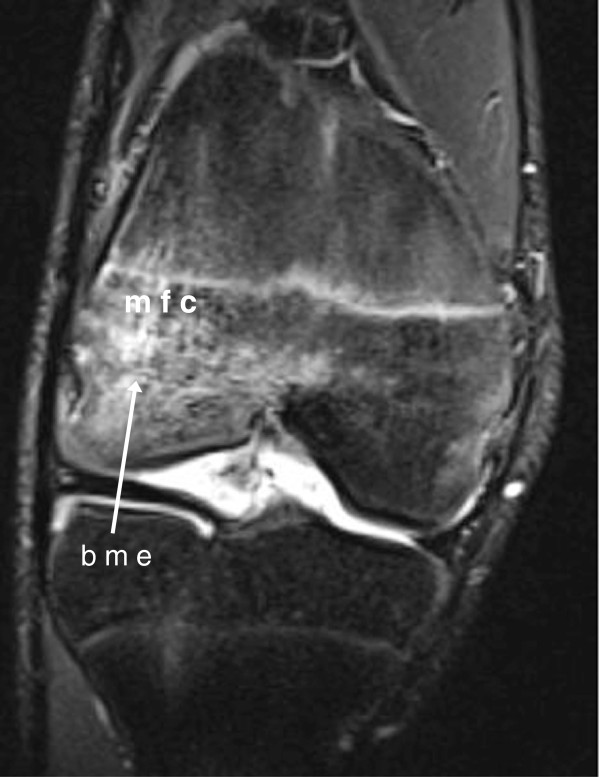
**Imaging of the symptomatic knee of a 14-year-old boy with JIA.** Coronal STIR MRI image showing bone marrow edema (bme) in the medial femoral condyle (mfc).

A median of 1 week (range 0–7 weeks) elapsed between the US and MRI examinations of the patients. No change of treatment in patients was initiated between the two imaging examinations. The number of pathological findings detected by the two methods in the 11 clinically affected joints are presented in Tables 2– 3 and 6. In the JIA patients, US revealed synovial hypertrophy in 22 areas of 11 joints, and there was concomitant synovial hyperemia in 19 (86%) of those areas (in 10 joints) (Figure 1A). MRI detected synovitis in 36 areas of 11 joints (Figure 1B). Effusion was identified by US in ten areas of six joints (Figure 2A) and by MRI in 15 areas of five joints (Figure 2B), and erosions were detected by US in two areas of two joints (one wrist and one ankle) and by MRI in five areas of three joints (three wrists) (Table 6). All erosions, detected by US or MRI, appeared in four of the patients: three with poly JIA and one with systemic onset type JIA with polyarticular course. Median duration of disease in those subjects was 3.8 years (range 0.2–8.5). MRI detected juxta-articular bone marrow edema in 16 areas of eight joints (five wrists, two knees, one ankle) (Figure 3).

**Table 6:** Anatomical location of erosions detected by MRI and US

	**MRI**	
**Joint (n = 3)**	**Area (n = 5)**	**Bone (n = 6)**
Wrist	radio-carpal	radius
	midcarpal	lunate, capitate
Wrist	midcarpal	hamate
Wrist	radio-carpal	lunate
	midcarpal	capitate
	**US**	
**Joint (n = 2)**	**Area (n = 2)**	**Bone (n = 2)**
Wrist	radio-carpal	radius
Ankle	talo-crural	talus

Conformity between the MRI and US findings in the areas examined in the 11 clinically affected joints is presented in Table 4.

### Healthy controls

**Figure 4:**
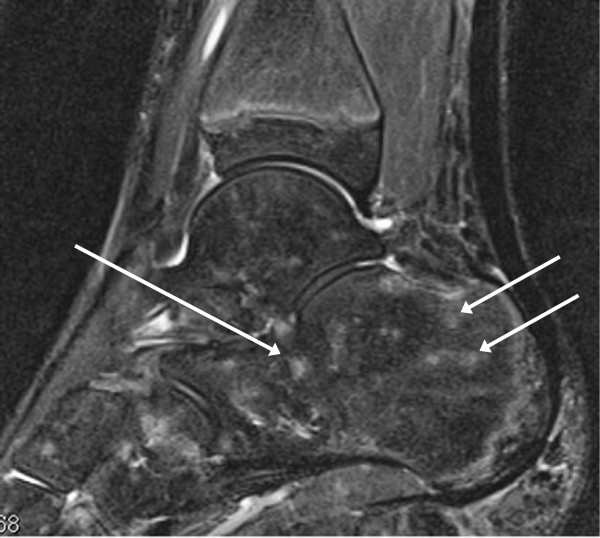
**Imaging of the asymptomatic ankle region of an 11-year-old healthy control.** A sagittal STIR MRI revealing multiple and patchy signal changes suggestive of foci of haematopoietic red marrow, or of focal bone marrow edema (arrows).

All US and MRI examinations of healthy controls were performed on the same day. In these individuals, slight effusion was detected in five joints (eight areas) by US and in six joints (14 areas) by MRI. No synovitis (synovial hypertrophy and/or synovial perfusion) or erosions were found in any of the controls. MRI revealed multiple and patchy, non-specific heterogeneous marrow signal changes on STIR images (Figure 4), in five joints (two ankles and three wrists) of six control subjects (median age 13 years, range 11–16 years).

Conformity between the MRI and US findings in the areas examined in eight joints of the healthy controls is presented in Table 5.

## Discussion

The synovial membrane is extremely thin in healthy children and visualization by US or MRI imaging may be difficult. Various systems involving quantitative or semi-quantitative methods are used to grade the synovial perfusion visualized by Doppler flow. Here, we assessed synovial perfusion according to the presence or absence of color Doppler flow. In our present study, Doppler flow was detected in 86% of the 22 diseased areas with synovial hypertrophy on US. Those observations are comparable to our previous findings, using the same definitions of synovial hyperemia, the same US equipment and Doppler settings, in the ankle (89%, [18]) and wrist (88%) regions [19]. Other studies in JIA patients have shown hyperemia in 93% of symptomatic MCP joints [20] and 77% of symptomatic knees [21]. The MRI RAMRIS definition of synovitis [16] used in our study, includes both synovial hypertrophy and increased synovial perfusion [22,23]. No synovial hypertrophy or synovial hyperemia was detected in any of the controls. This agrees with a recent US study of JIA patients showing that any presence of Doppler flow was significantly associated with clinical synovitis, and that Doppler flow was absent in all the healthy controls [17].

The differentiation of active synovial thickening from joint effusion may be difficult with non-enhanced MR imaging. Physiological joint effusion is common in healthy children, but there is no consensus concerning the normal amount of synovial effusion in healthy individuals. Indeed, a recent MRI study [24] detected fluid in the wrists of children at a relatively large volume that has previously been considered to be pathological in adults [25]. In our investigation, effusion was the only finding in the healthy controls, as shown by US in five joints and by MRI in six joints.

**Figure 5:**
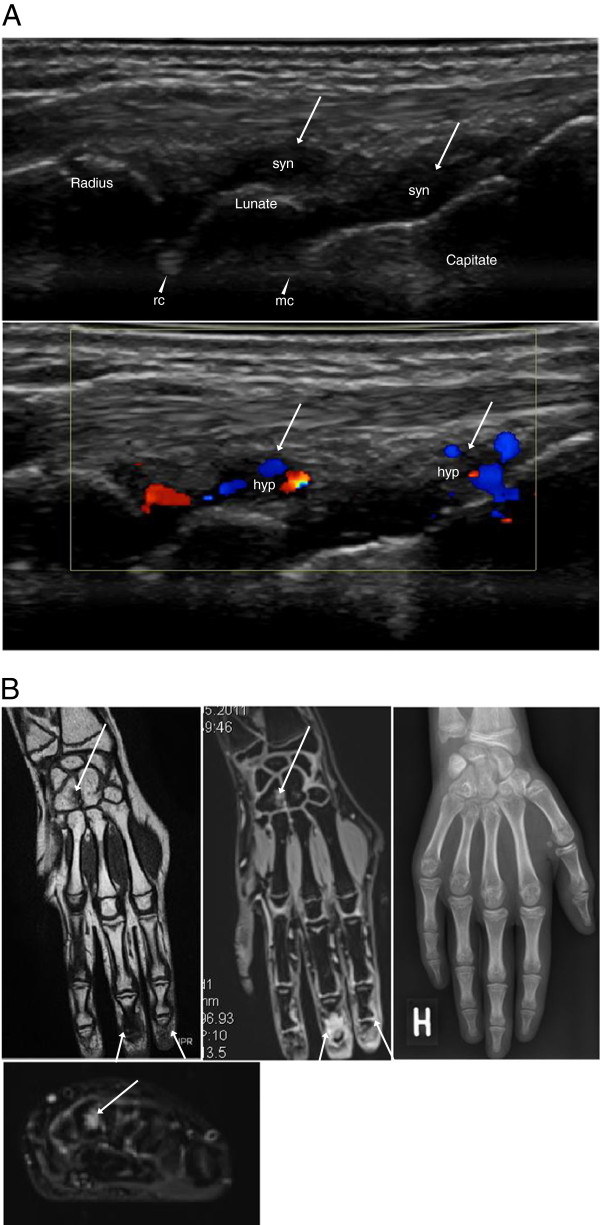
**Imaging of the symptomatic wrist region of a 13-year-old girl with JIA.** (**A**) Dorsal sagittal US scans of the radiocarpal (arrowhead, rc) and midcarpal (arrowhead, mc) joints showing synovial hypertrophy (syn, top image), with hyperemia (hyp) on color Doppler (bottom image), in the dorsal recesses (arrows). There are no visible erosions. (**B**) Overview of pathology in coronal MRIs of the hand and wrist. An erosion in the Hamate (arrow) and synovitis in the 2nd and 3rd DIP joints (arrows) are seen both in the coronal T1 SE image to the left, and in the coronal postcontrast 3D T1 GRE VIBE sequence in the middle. The axial STIR image (bottom image) reveals the bone marrow edema (arrow) surrounding the erosion of the Hamate. No pathology is displayed in the concurrent, posteroanterior X-ray (right image).

MRI proved to be the best method for identifying erosions in JIA patients (Figure 5A + B), which agrees with the results of previous studies of patients diagnosed with rheumatoid arthritis (RA) [26] or JIA [27]. MRI detected erosions in five areas of three wrist regions (Table 6). Three of these erosions were detected in anatomical locations that are not accessible to US waves. However, these areas are also difficult to evaluate with MRI and, due to their anatomic peculiarities, they are associated with an intrinsically higher risk of being scored as false positive [28]. Furthermore, a recent MRI study of healthy children demonstrated a high prevalence of bony, erosion-like carpal depressions that increased with age in the normal skeleton of the hand [23], and MRI investigations have revealed numerous changes of the same type in the wrists of healthy adults, primarily in the capitate and lunate bones [29,30]. 

An MRI finding of bone marrow edema predicts erosive disease in RA patients [31-33], whereas the prognostic value of such edema has not yet been established in JIA. A major advantage of MRI over US consists of detecting pathology in locations that are not accessible to US waves, such as the bone marrow. In our study, MRI revealed signs of juxta-articular bone marrow edema in six of the 10 JIA patients (four with poly JIA, one with systemic onset type JIA with polyarticular course, and one with psoriasis arthritis). Bone marrow edema is either rare or absent in healthy adults [30]. In a previous small MRI pilot study of the iliac crest (Laurell, unpublished data), we found that bone marrow edema associated with normal epiphyseal growth in healthy young individuals was difficult to distinguish from the pathological edema caused by enthesitis in JIA patients. Furthermore, other pediatric studies have reported similar MRI findings of physiological edema in healthy individuals at the iliac crest [34], in the wrist region [24], and in the ankle region [35,36]. In the present investigation, MRI revealed multiple and patchy heterogeneous marrow signal changes on STIR images in five joints of four control subjects, suggestive of isolated foci of residual haematopoietic red marrow, or of focal bone marrow edema. Consequently, it may be difficult to use MRI to detect pathological bone marrow edema in children and adolescents.

There are no validated MRI or US scoring systems for the assessment of inflammatory and joint damage abnormalities in JIA, and there is little knowledge of the normal US and MRI reference values of each joint at different developmental stages in children. It is possible that a large part of the knowledge obtained in studies in adult rheumatology can be applied to children as well, but the imaging techniques must be further validated in all fields of pediatric rheumatology. In our study we adopted the OMERACT [15] definitions for US pathology, and the RAMRIS definitions [16] for MRI pathology. The MRI RAMRIS system, designed for use in adults, has been validated in the wrist and MCP joints of adult subjects, and was recently also tested on wrists in a pediatric population.

For assessment of children, US offers specific advantages over MRI in that it is non-invasive, does not require sedation or general anesthesia (which facilitates repeated examinations for follow-up), is quickly accessible bedside, and is easy to combine with clinical assessment (interactivity). On the other hand, MRI gives an overview and also detects pathology in locations that are not accessible to US waves. This last mentioned factor might explain some of the differences between MRI and US results in our study evaluating complex joint structures like the wrist regions and ankle regions. Advantages and disadvantages of MRI and US, respectively, are summarized in Table 7. In our study the positioning of joints also differed slightly between the MRI and US examinations, which might have influenced the distribution of effusion on MRI and US images, respectively. On MRI images intravenous contrast is required to differentiate synovial tissue from effusion; the differentiation is easily performed on the fat-saturated post-contrast MRI sequences. The need for intravenous contrast injection is a disadvantage in dealing with pediatric patients. On US images, synovial tissue is hypoechoic and effusion is generally anechoic, but the differentiation is not always easy and confusion might occur as fluid may be very hypoechoic, nearly anechoic, and effusion containing particles may be hypoechoic. In anatomical areas where compression with the transducer is easily performed, e.g. the suprapatellar recess, differentiation between synovial tissue and effusion is facilitated (Figure 2A). Doppler US examination, displaying vascularization, and dynamic US examination, during active or passive mobilization of the soft tissues examined, may also facilitate such differentiation. In children, the hypoechoic synovial tissue might also be difficult to distinguish from the hypoechoic cartilage of the epiphyses. Doppler examination is generally not a solution to this problem, since vascularization can be present in both hypertrophic synovial membranes and cartilaginous epiphyses during growth. Therefore, to avoid diagnostic errors, it is important to have good knowledge of the normal US appearance of each joint at different stages of development, and it is also imperative to use a meticulous scanning technique that allows clear interpretation of possible anisotropic artifacts [37] (Laurell, 2012, Clinical and Experimental Rheumatology, accepted for publication). 

**Table 7:** US versus MRI in JIA

** US**	** MRI**
Early diagnosis	Early diagnosis
Soft tissues	Soft tissues
Hyperemia/Doppler	Hyperemia/contrast enhancement
Available at point of care	Poor availability
Non-invasive	IV contrast
	Sedation/general anesthesia in young children
Quick	Time consuming
Focal imaging	Overview
Bone surface only	Bone, bone marrow edema
Machine and operator dependent	Machine and operator dependent
Limited normative data	Limited normative data
Not validated	Not validated

In our study two small and flat Baker’s cysts with a thin synovial wall were visualized on fat-saturated T1w post-contrast MRI sequences, but were not detected on US. US is more sensitive than MRI in detecting small volumes of effusion, but in these cases no fluid content, or hyperemia of the synovial tissue, could be visualized on Doppler US. The reason for the discrepancy between the two imaging modalities in detecting tendon sheath involvement is unclear. It can be essential to apply as little pressure as possible with the US transducer during the examination of superficial tendons in order to visualize vascularization on Doppler examination, and synovial hypertrophy might be difficult to distinguish from effusion within a tendon sheath.

A main disadvantages of MRI, is that it requires sedation of young children, an age group with a high prevalence of JIA [38]. In our study, a 6-year-old patient had to be excluded, because the age-matched control failed to remain still during the MRI procedure, while US examinations could be performed on both individuals without any objections.

The present investigation was descriptive in nature and was not designed to compare the results of clinical assessment and imaging, but rather to compare two imaging modalities. Four different anatomical areas were assessed in the knee and ankle region, respectively, and three different areas in the wrist region. The objective was to investigate whether the US and MRI findings, respectively, occur in the same or in different anatomical locations. The inclusion criterion was being a child with clinically active arthritis. Patients without focal clinical symptoms were not presented to the US examiner, who was also blinded to other aspects of the children, such as clinical status and subtype. As the examinations on patients and controls were performed at different occasions and at different locations the examiner was not blinded to whether he was evaluating JIA patients or controls. Another experienced musculoskeletal radiologist analyzed all MRI images. He was blinded to the results of US examinations but, as the dates of examination were differing between the two groups, not to the respective subject category (patients or controls), which might have constituted a bias.

Another study has previously examined the role of MRI versus US in assessing knee inflammation in JIA [9], but to our knowledge, our pilot study is the first to compare results of MRI and Doppler US for the overall assessment of all aspects of JIA (i.e., considering synovial hypertrophy, synovial perfusion/enhancement, effusion, bone erosions, and bone edema). A weakness of the present study is that the US assessments and the MRI examinations, respectively, were evaluated for accuracy by only one experienced musculoskeletal radiologist. Furthermore, the discrepancies we observed between the US and MRI findings might have been related to the time span between the two examinations, as well as to the fact that most of the patients had received pharmacological treatments with a potential effect on synovitis. The US examination protocol used in our study did not include the radio-ulnar joint and hence we cannot rule out any synovitis in this area of the wrist, but MRI showed involvement of this joint in 5 out of 6 wrists examined (Table 3). Accordingly, in future investigations of US examination of the wrist in JIA, we will use a revised and more appropriate scanning protocol that also includes the radio-ulnar joint.

## Conclusions

Although small, this study has yielded results indicating that both MRI and US provide valuable imaging data on disease activity in various joints of children with JIA. Importantly, the two techniques seem to complement each other and give partly different information on the patients who are assessed. In JIA, as well as in adult rheumatology, US seems to provide useful imaging information that could make it an option, on many occasions, in daily clinical practice. However, it should be pointed out that the current work represents a pilot study, and thus our results need to be confirmed in a larger prospective clinical investigation.

## Competing interests

The authors declare that they have no competing interests.

## Authors’ contributions

LL participated in design of the study, performance of clinical examinations, acquisition of data, statistical analysis, and was responsible for analysis of the results and drafting of the manuscript. MCP was involved in design of the study, analysis of the US results, performance of US examinations, comparison of imaging modalities, and drafting of the manuscript. SN helped design the study, perform clinical examinations, and revise the manuscript. MZ contributed to design the study, the performance of clinical examinations, and revision of the manuscript. MB helped design the study, was responsible for analysis of the MRI results, and helped compare the imaging modalities and draft the manuscript. AF helped design the study, analyze the results, and draft the manuscript. All authors read and approved the final manuscript.

## Supplementary Material

Additional file 1 Detailed descriptions of MRI protocols are included in ‘additional file’. Click here for file
