# Supplementary material for: Comparison of ultrasonography with Doppler and MRI for assessment of disease activity in juvenile idiopathic arthritis: a pilot study
Source: Pediatr Rheumatol Online J. 2012 Aug 16;10:23. doi: 10.1186/1546-0096-10-23 (PMC3608365; doi:10.1186/1546-0096-10-23)
Supplement: Additional file 1 — Detailed descriptions of MRI protocols are included in ‘additional file’. [file 1546-0096-10-23-S1.pdf]

The following MRI protocols were applied in patients and healthy controls, except that no Gadolinium contrast (Magnevist, Bayer, Germany) was administered to the controls:

Wrists

Gradient echo scout (slice thickness [ST] 6 mm, field of view [FOV] 400 x 400 mm, TE 3.69 milliseconds [ms], TR 7.8 ms, scan time 17 s); coronal T1 weighted (T1W) turbo spin echo (TSE) (ST 1.5 mm, FOV 250 x 250 mm, matrix resolution 0.3 x 0.3 x 1.5 mm, TE 25 ms, TR 832 ms, scan time 4 min 28 s); coronal STIR (ST 2.5 mm, FOV 180 x 180 mm, matrix resolution 0.9 x 0.8 x 2.5 mm, TI 220 ms, TE 32 ms, TR 4500 ms, scan time 2 min 48 s); fat-saturated (FS) T2w (3T Trio) (ST 3 mm, FOV 120 x120 mm, matrix resolution 0.4 x 0.4 x 3 mm, TE 94 ms, TR 3250 ms, scan time 4 min 20 s); axial STIR covering wrist and MCP joints (ST 5 mm ST, FOV 180 x 180mm, matrix resolution 0.6 x 0.6 x 5mm, TI 220 ms, TE 32 ms, TR 4500 ms, scan time 2 min 30 sec); FS T2w TSE covering wrist and MCP joints (ST 5 mm, FOV 120 x 120 mm, matrix resolution 0.4 x 0.4 x5 mm, TE 94 ms, TR 3250 ms, scan time 4 min 0 s); gradient echo 3D T1w VIBE (ST 0.9 mm, FOV 250 x 250 mm, matrix resolution 0.9 x 0.9 x 0.9mm, flip angle [FA] 10 degrees, TE 6 ms, TR 13.5 ms, scan time 2 min 35 s). After intravenous injection of 0.1 ml/kg body weight gadolinium contrast, the T1-weighted sequences were repeated. Total imaging time varied between 25 and 35 min.

Knees

Gradient echo scout (ST 8 mm, FOV 400 x 400 mm, TE 3.69 ms, TR 7.8 ms, scan time 17 s); coronal and axial T1w TSE (ST 3.5 mm, FOV 150 x 150 mm, matrix resolution 0.6 x 0.5 x 3.5 mm, TE 17 ms, TR 790 ms, scan time 2 min 24 s); coronal and sagittal STIR (ST 3 mm, FOV 160 x 160 mm, matrix resolution 0.7 x 0.6 x 3 mm, TI 220 ms, TE 34 ms, TR 4350 ms, scan time 1min 29 s); sagittal 3D proton density-weighted (PDW) FS TSE SPACE (3T Verio

scanner only) (ST 0.6 mm ST, FOV 160 x 160 mm, matrix resolution 0.6 x 0.5 x 0.6 mm, TE 44 ms, TR 1000 ms, scan time 9 min 26 s); sagittal gradient echo 3D T1w VIBE (ST 0.6 mm, FOV 160 x 160 mm, matrix resolution 0.6 x 0.6 x 0.6 mm, FA 10 degrees, TE 5.39 ms, TR 11.6 ms, scan time 3 min 19 s). After intravenous injection of 0.1 ml/kg body weight gadolinium contrast, the T1-weighted sequences were repeated. Total imaging time varied between 30 and 40 min.

Ankles

Gradient echo scout (ST 8 mm, FOV 400 x 400 mm, TE 3.69 ms, TR 7.8 ms, scan time 11 s); coronal, axial, and sagittal T1w TSE (ST 3 mm, FOV 150 x 150 mm, matrix resolution 0.5 x 0.4 x3 mm, TE 13 ms, TR 5540 ms, scan time between 1 min 54 s and 3 min 5 s); coronal and sagittal STIR (ST 3 mm, FOV 160 x 160 mm, matrix resolution 0.5 x 0.5 x 3 mm, TI 220 ms, TE 36 ms, TR 4500 ms, scan time 2 min 48 s); sagittal gradient echo 3D T1w VIBE (ST 0.7 mm, FOV 160 x 140 mm, matrix resolution 0.9 x 0.7 x 0.7 mm, FA 15 degrees, TE 1.96 ms, TR 5.68 ms, scan time 4 min 40 s). After intravenous injection of 0.1 ml/kg body weight gadolinium contrast, the T1-weighted sequences were repeated. Total imaging time varied between 30 and 40 min.
